# Albuminuria during treatment with angiotensin type II receptor blocker is a predictor for GFR decline among non-diabetic hypertensive CKD patients

**DOI:** 10.1371/journal.pone.0202676

**Published:** 2018-08-27

**Authors:** Mi-Yeon Yu, Dong Ki Kim, Jung Hwan Park, Sung Joon Shin, Sang Ho Lee, Bum Soon Choi, Chun Soo Lim, Ho Jun Chin

**Affiliations:** 1 Department of Internal Medicine, Hanyang University Guri Hospital, Guri, South Korea; 2 Department of Internal Medicine, Seoul National University Hospital, Seoul, South Korea; 3 Department of Internal Medicine, Seoul National University College of Medicine, Seoul, South Korea; 4 Department of Internal Medicine, Konkuk University School of Medicine, Seoul, South Korea; 5 Department of Internal Medicine, Dongguk University Ilsan Hospital, Goyang, South Korea; 6 Department of Internal Medicine, Kyung Hee University Medical Center, Seoul, South Korea; 7 Department of Internal Medicine, Seoul St Mary’s Hospital, Seoul, South Korea; 8 Department of Internal Medicine, Seoul National University Boramae Medical Center, Seoul, South Korea; 9 Department of Internal Medicine, Seoul National University Bundang Hospital, Seong-Nam, South Korea; 10 Research Institute of Salt and Health, Seoul, South Korea; Universita degli Studi di Perugia, ITALY

## Abstract

**Background:**

Albuminuria is a predictor of disease progression in patients with chronic kidney disease (CKD). However, the ability of proteinuria parameters measured at various time periods to predict renal outcomes is unclear.

**Method:**

This observational cohort study included 165 non-diabetic hypertensive CKD patients who took olmesartan medoxomil. We measured the albuminuria at five different time points (0, 2, 4, 26, and 38 months) and the mean levels. The mean albuminuria levels were calculated during 0–4 months, 0–26 months, and 0–38 months. The renal outcome was defined as a decline in eGFR ≥ 40% during the entire study period.

**Result:**

The albuminuria at five different time points and the mean albuminuria levels were independent risk factors for a worse renal outcome after adjusting for age, sex, and estimated glomerular filtration rate (eGFR) at enrollment and were able to predict the renal outcome, although the performance of the estimation tended to be more effective using the mean albuminuria level at the 38-month follow-up time point. The risk of a decline in eGFR ≥ 40% was increased by 1.690-folds [95% CI 1.110–2.572, *P* = 0.014] per 500 mg/day increase in the mean albuminuria at 38 months. With a cut-off value of 897 mg/day for mean albuminuria at 38 months after treatment, a decline in eGFR ≥ 40% was predicted with a sensitivity of 88.9% and specificity of 81.3%. The ability of albuminuria to predict a renal event at different measurement points does not differ in CKD patients.

**Conclusion:**

The time-averaged albuminuria cut-off of 900 mg/day during the 3-year follow-up period showed high sensitivity and specificity for predicting a decline in eGFR ≥ 40% in CKD patients, although the albuminuria at different measurement points did not predict a worse renal outcome.

## Introduction

Proteinuria, usually referred to as albuminuria, is a strong predictor of disease progression in patients with chronic kidney disease (CKD), regardless of the presence of diabetes mellitus [1–7]. In diabetic CKD patients, baseline proteinuria, changes in proteinuria, and residual proteinuria at 6 months after treatment with a renin-angiotensin-aldosterone system inhibitor (RASi) predicated worse renal outcomes [2, 8, 9]. The mean proteinuria level during follow-up (time-averaged proteinuria [TA-p]) is reportedly a strong marker of disease activity and response to treatment in immunoglobulin A (IgA) nephropathy and non-diabetic CKD patients [1, 3, 4, 10]. Several reports have suggested that TA-p is a more important prognostic factor than the baseline proteinuria level and changes in proteinuria levels during short-term follow-up [1, 4, 10] Change in proteinuria is also reported as a more important risk factor for renal outcome than baseline proteinuria [3, 10]. However, there are several limitations to previous reports in terms of the ability of proteinuria to predict renal outcomes in CKD patients. Most studies on this topic involved data of patients with diabetic nephropathy or IgA nephropathy [1, 2, 8–11]. CKD includes heterogeneous diseases with unique pathophysiology, although it might have common pathways involved in disease progression and the effects of proteinuria on renal outcomes in CKD would differ from that in a single category of renal disease, such as diabetic nephropathy or IgA nephropathy [12]. Therefore, more data about CKD alone are needed. Furthermore, which one among baseline proteinuria, changes in proteinuria, residual proteinuria, or TA-p during the follow-up period mediates the progression of renal disease is unclear, and the extent of the probability of inducing a worse renal outcome by proteinuria is also undetermined in CKD [12]. In addition, albuminuria rather than proteinuria in CKD is recommended for diagnosis and follow-up, but most previous studies used proteinuria as a predictor of renal outcome [13, 14]. The other limitation in previous studies is statistical weakness. Baseline proteinuria may influence changes in proteinuria, residual proteinuria, and TA-p during the follow-up period. Previous reports included multivariate models of baseline proteinuria and other related factors, which cannot exclude the interaction among proteinuria parameters.

Therefore, we investigated the predictability of albuminuria for renal outcome using various related parameters and compared the priority to predict renal outcome among proteinuria-related parameters in a non-diabetic hypertensive CKD cohort. We analyzed the influence of repeated measurements of albuminuria on renal outcomes using the generalized estimating equations (GEE) to avoid interactions among parameters of proteinuria and compared the receiver operating characteristic (ROC) areas to determine the predictability for a renal outcome by parameters of proteinuria.

## Materials and methods

### Study population

This study included participants recruited from a previous open-label, case-control, randomized clinical trial (clinicaltrials.gov registration number NCT01552954, posted at 13/03/2012). The inclusion criteria were described in the previous report [15]. Briefly, the participants had hypertension, eGFR ≥ 30 ml/min/1.73 m^2^, random urine albumin-to-creatinine ratio ≥ 30 mg/g creatinine more than two times with a ≥ 1 week interval in the last 6 months, and an ability and willingness to provide written informed consent. Patients were selected from the outpatient renal clinics of seven centers in Korea. In the trial phase, all participants were receiving an angiotensin type II receptor blocker (ARB) at random assignment to either intensive low salt diet education or conventional education. Participants followed an educational program for 8 weeks after randomization and then laboratory tests to assess proteinuria and 24-hour urine sodium, urea, potassium, and creatinine clearance were performed. The trial phase started on March 2012, and finished on March 2013. After the trial, all participants had regular follow-up at out-patient clinics and were asked to participate in the cohort phase on September 2014.

### Ethics statement

All clinical investigations were conducted in accordance with the 2008 Declaration of Helsinki and the guidelines of good clinical practice. Informed written consent was obtained from each patient before inclusion. The study protocol has been approved by the Seoul National Univserity Bundang Hospital institutional review board (IRB number: B-1405/251-007).

### Study protocol

**Fig 1:**
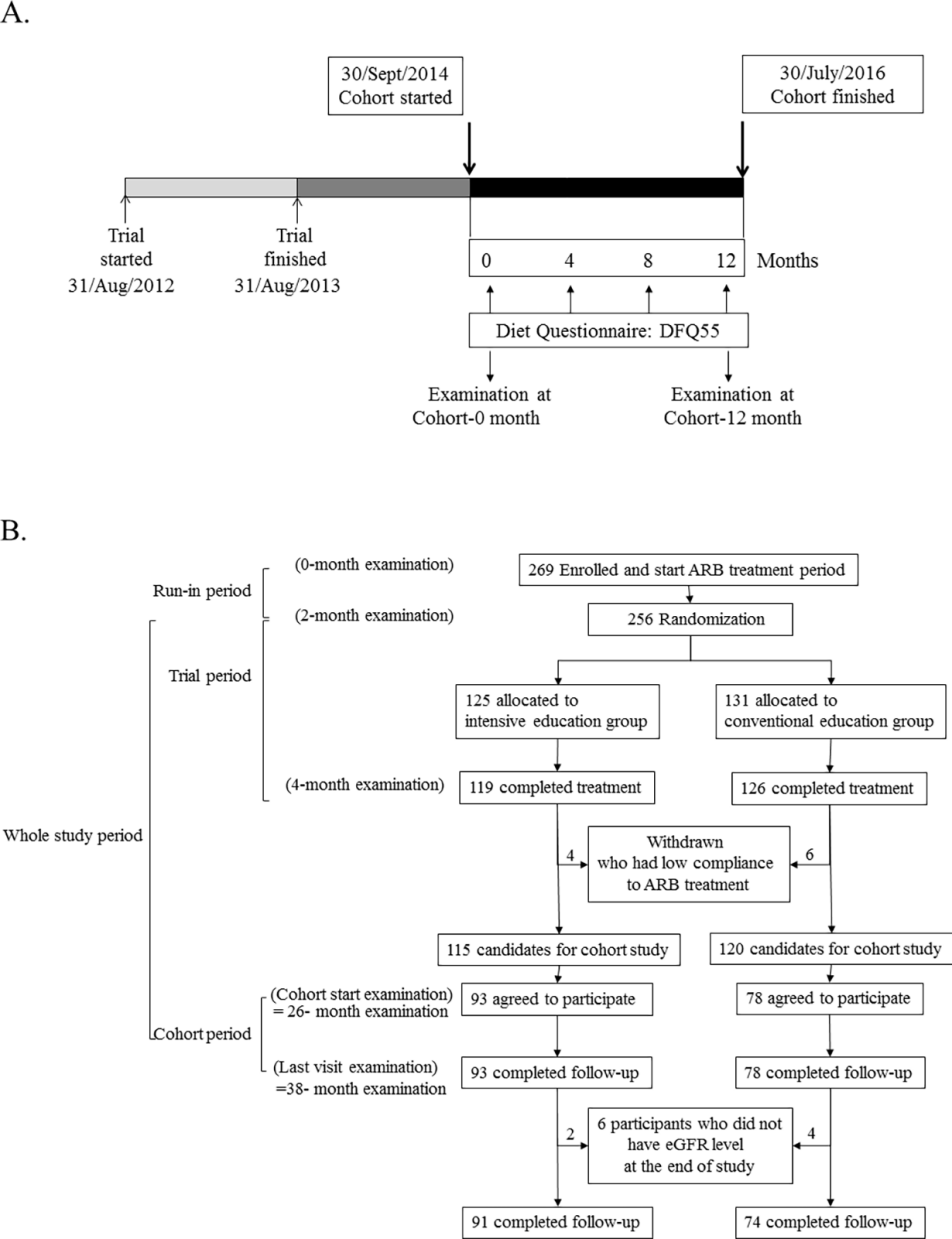
Schema of this study. A. Time-line for trial phase and cohort phase. The trial phase started on August 31, 2012 and ended on August 31, 2013. The cohort study was initiated on September 30, 2014 and ended on July 30, 2016. Examinations for demographic findings and laboratory tests were conducted at the first and last visit during the cohort phase. During the cohort phase, diet education was delivered to all patients for 20 minutes per session through a telephone call with a clinical dietitian every 4 months. B. Study flow chart. Among participants in the trial phase, we excluded 10 whose compliance to angiotensin type II receptor blocker medication was <60%. We enrolled 165 patients who completed the cohort phase and laboratory tests of estimated glomerular filtration rate.

The protocol of the trial phase has been described elsewhere [15]. The protocol included a run-in period for antihypertensive medication adjustment. All of the participants had to stop all RASi or diuretic agent use and switch to different categories of antihypertensive medications. After an 8-week run-in period, the participants were subjected to baseline (0-week) laboratory testing and took olmesartan medoxomil (one 40-mg tablet/day; Daewoong Pharmaceutical Co. Ltd./Daiichi Sankyo Korea Co. Ltd., Seoul, South Korea) until the end of the study. After another 8 weeks, the participants were randomly assigned to an intensive low-salt diet education or conventional education program after the second (8-week) laboratory examination. The intensive education group was closely supported by a clinical dietician and telephone feedback for 30 minutes one time per week for 8 weeks. The target daily sodium intake was <100 mEq in the intensive education group, while a ≥25% reduction in salt intake was recommended in both groups. After 8 weeks of educational intervention, the participants underwent a third (16-week) laboratory examination. We assessed drug compliance using pill counts. We calculated estimated daily creatinine excretion using Tanaka’s equation (-2.04 × age + 14.89 × body weight [kg] + 16.14 × height [cm]–2244.45 mg/day] [16], the performance of which was confirmed [17]. To eliminate errors from the incompleteness of the 24-hour urine collection and difference in muscle mass, we multiplied the 24-hour urine variables-to-creatinine ratio (a unit/g creatinine) by estimated daily creatinine excretion (g/24 hours) and obtained the estimated urine excretion of a 24-hour urine variable (in a unit/24 hours) [17]. Estimated protein intake was calculated as follows: (estimated 24-h urine urea nitrogen [g/day] + body weight [kg] × 0.031 × 6.25 [g protein/day] normalized by body weight [g protein/kg body weight/day]). We started to enroll participants for the cohort phase at 26.8 ± 4.0 months after enrollment of the trial phase (Fig 1). Of the 245 participants who finished the trial phase, we excluded 10 from the cohort phase because of poor compliance (<60%) with ARB medications during the trial phase. At this stage, participants provided written informed consent for this study and underwent a fourth laboratory examination (26-month examination). All participants in the cohort phase were given dietary support by the same clinical dietician with the same protocol as the trial phase in the form of a 30-min telephone call once every 4 months during the 12-month study period. After the 12-month cohort phase, participants visited the clinic for a fifth laboratory examination mean 38.1 ± 4.1 months after enrollment for the trial phase (38-month examination). During the cohort phase, participants were followed at the outpatient clinic as directed by the researcher. We selected 165 participants who finished the cohort study and had eGFR values at 0 weeks and 38 months into the follow-up period. There was no change of study protocol after trial commencement.

### Measurements and the renal outcome

We evaluated the predictability of albuminuria at each visit and the mean albuminuria during the defined period to predict a renal outcome. The mean albuminuria levels were calculated during 0–16 weeks, 0 weeks to 26 months, and 0 weeks to 38 months. The percent change of albuminuria or eGFR was calculated as follows: (last level—initial level) × 100/initial level (%). The renal outcome was defined as a decline in eGFR ≥ 40% during the entire study period. The eGFR was estimated by the Modification of Diet for Renal Disease (MDRD) equation using isotope dilution mass spectrometry–traceable creatinine, which was previously validated in Koreans [18, 19].

### Statistical analysis

The analyses were performed using SPSS statistics (version 22.0; IBM Corporation, Armonk, NY, USA). Continuous variables are expressed as mean ± SD and categorical variables are expressed as frequency (percentage). An independent Student’s *t* test was used to compare continuous variables between the two subgroups. Pearson’s chi-square test or Fisher’s exact test was used to analyze the categorical variables according to the probability. Wilcoxon signed rank test was used to compare two albuminuria values performed at different times. To assess the difference in repeatedly measured albuminuria over time according to the presence of a decline in eGFR ≥ 40%, we used the repeated measures analysis of variance (ANOVA) test with Bonferroni correction. The correlation between renal outcome and other factors was estimated with Pearson’s correlation coefficient and tested with multiple binomial logistic regression analysis for albuminuria and renal outcome by adjustment for age, sex, and related factors such as smoking habit, pulse rate, usage of steroids, eGFR level during the enrollment period of the trial phase, randomization to diet education, and mean sodium intake during the entire study period. The Hosmer-Lemeshow goodness-of-fit test were used to evaluate the model fit. We searched the effect of repeatedly measured albuminuria to predict the renal outcome with the GEE adjusted for age, sex, and independent factors related to the outcome. The ROC was evaluated for the parameters of albuminuria measured at various time points to predict the renal outcome. We compared the equality of ROC area of each albuminuria variable to predict the renal outcome using the STATA program (version 14; Statacorp LLC, College Station, TX, USA). Differences with two-tailed P values < 0.05 were considered statistically significant.

## Results

### Patient characteristics

The non-participants in this study were more frequently allocated to the intensive diet education group and had lower compliance to olmesartan medoxomil during the trial phase (S1 Table). Other findings of demographics, lifestyle, and laboratory measurements did not differ between participants and non-participants (S1 Table). The mean age of participants was 50.0 years. There were 79 men. During the enrollment period of the trial phase, mean eGFR was 67.1 ± 24.7 mL/min/1.73 m^2^. The mean values of serum creatinine and albuminuria were 1.14 mg/dL (SD ± 0.40) and 1061 mg/day (SD ± 1094) (S1 Table). The albuminuria level values including mean, minimum, maximum, for all the visits were provided in S2 table. At the last visit of the cohort phase (38-month examination), an angiotensinogen converting enzyme inhibitor (ACEI) was used in 12 (7.3%) patients and an ARB was used in 137 (83.0%) patients. Overall, 149 (90.3%) patients had taken an ACEI or an ARB. The mean albuminuria level was 643 mg/day at the last follow-up (38-month examination), lower than that at the enrollment period of the trial phase (p value by Wilcoxon signed rank test, <0.001). A 40% decline in eGFR was significantly related to the albuminuria measured at 0-week (r = 0.257; p = 0.001), 8-week (r = 0.252; p = 0.001), 16-week (r = 0.175; p = 0.023), 26-month (r = 0.415; p < 0.001), and 36-month (r = 0.195; p = 0.014), the mean albuminuria measured from the 0-week to the 16-week (r = 0.253; p = 0.001), from the 0-week to the 26-month (r = 0.314; p < 0.001), and from the 0-week to the 36-month (r = 0.349; p < 0.001), and initial eGFR (r = -0.171; p = 0.025). In repeated measures ANOVA including five measurements of albuminuria at each visit and three mean albuminuria values at three different time periods, repeatedly measured albuminuria levels of the participants with a decrease of eGFR ≥ 40% were higher than the albuminuria levels of participants without a renal outcome during the entire study period (p = 0.005; Table 1). The mean percent change of eGFR was -8.3% (SD ± 20.0, mean eGFR slope was -1.4 mL/min/1.73 m^2^/year (SD ± 4.5), and mean percent change of albuminuria was -5.3% (SD ± 146.5) during the entire study period (0–38 months). Nine (5.5%) participants reached the renal outcome, and 34 (20.6%) participants had a decline of eGFR slope ≥ 4 mL/min/1.73 m^2^/year.

**Table 1:** Difference of albuminuria during study period according to the change of eGFR.

	Decline of eGFR		
	< 40%	≥ 40%	P value
Albuminuria (mg/day, mean ± SD)			
0-week	995 ± 1050	2171 ± 1621	0.003
8-week	549 ± 790	1032 ± 667	0.092
16-week	452 ± 710	879 ± 671	0.099
26-month	533 ± 693	1481 ± 928	<0.001
38-month	606 ± 916	1425 ± 1109	0.016
Averaged albuminuria (mg/day, mean ± SD)			
0-week to 16-week	665 ± 796	1361 ± 829	0.018
0-week to 26-month	632 ± 740	1391 ± 736	0.005
0-week to 38-month	520 ± 594	1191 ± 603	0.002

Variables were expressed as mean ± standard deviation.

The repeatedly measured albuminuria among participants with a decline of eGFR ≥ 40% was higher than that among participants without a renal outcome during the entire study period in a repeated-measures analysis of variance (ANOVA) test (p = 0.005).

A repeated-measures ANOVA test with Bonferroni correction was used to assess the difference in repeatedly measured albuminuria between two groups (with/without a 40% decline in eGFR) at each period.

Decline of eGFR, participants were grouped according to decline of eGFR; 40%, compared eGFR at the end of the cohort phase to eGFR at the enrollment period of the trial phase; eGFR, estimated glomerular filtration rate calculated by the MDRD equation using IDMS-traceable serum creatinine; 0-week, albuminuria measured at the initiation of the trial phase; 8-week, albuminuria measured at 8 weeks after initiation of the trial phase; 16-week, albuminuria measured at 16 weeks after initiation of the trial phase; 26-month, albuminuria measured at the enrollment period of the cohort phase; 38-month, albuminuria measured at the end of the cohort phase; 0-week to 16-week, mean albuminuria measured from the 0-week to the 16-week period of the trial phase; 0-week to 26-month, mean albuminuria measured from the 0-week of the trial phase to the enrollment period of the cohort phase; 0-week to 38-month, mean albuminuria level throughout the entire study period. eGFR, estimated glomerular filtration rate.

### Effects of measurements of albuminuria on a 40% decline in eGFR

We analyzed the independent risk factors to estimate a 40% decline in eGFR with multiple binomial logistic regression analysis (Table 2). We modeled the regression with each albuminuria value or mean albuminuria value at different time points, ages, sexes, and other related factors to the renal outcome such as smoking habit, pulse rate, usage of steroids, eGFR level during the enrollment period of the trial phase, randomization to diet education, and mean sodium intake during the entire study period. The values of albuminuria at 0 weeks, 26 months, and 38 months and three mean albuminuria values were significant factors for predicting the renal outcome. For example, the risk of a decline in eGFR ≥ 40% was increased by 1.690-fold (95% confidence interval, 1.110–2.572-fold, p = 0.014) per 500 mg/day increase in mean albuminuria at 38 months. The other independent risk factor was eGFR during the enrollment period on multiple logistic regression.

**Table 2:** Estimated relative risk for decline of eGFR ≥ 40% according to the levels of albuminuria at various time points.

	B	S.E.	Wald	RR	95% CI for RR		P value
Albuminuria (per 500 mg/day)							
0-week	0.314	0.134	5.500	1.369	1.053	1.781	0.019
8-week	uc	uc	uc	uc	uc	uc	0.640
16-week	uc	uc	uc	uc	uc	uc	0.599
26-month	0.549	0.193	8.115	1.732	1.187	2.527	0.004
38-month	0.243	0.138	3.103	1.275	0.973	1.669	0.078
Averaged albuminuria (per 500 mg/day)							
0-week to 16-week	0.328	0.161	4.142	1.389	1.012	1.905	0.042
0-week to 26-month	0.401	0.173	5.350	1.494	1.063	2.099	0.021
0-week to 38-month	0.525	0.214	5.992	1.690	1.110	2.572	0.014

eGFR, estimated glomerular filtration rate; S.E., standard error; RR, relative risk; CI, confidence interval; uc, unable to calculate.

Each model by the multiple binomial logistic regression analysis was adjusted for age, sex, and factors related to the risk of a decline of eGFR ≥ 40% such as smoking habit, pulse rate, steroid usage, eGFR at the enrollment period of the trial phase, randomization to diet education, and mean sodium intake during the entire study period.

0-week, albuminuria measured at the initiation of the trial phase; 8-week, albuminuria measured at 8 weeks after initiation of the trial phase; 16-week, albuminuria measured at 16 weeks after initiation of the trial phase; 26-month, albuminuria measured at the enrollment period of the cohort phase; 38-month, albuminuria measured at the end of the cohort phase; 0-week to 16-week, mean albuminuria measured from the 0-week to the 16-week period of the trial phase; 0-week to 26-month, mean albuminuria measured from the 0-week of the trial phase to the enrollment period of the cohort phase; 0-week to 38-month, mean albuminuria level throughout the entire study period.

We confirmed that higher levels of repeated albuminuria measurements predicted a higher-risk renal outcome by the GEE model adjusted for age, sex, and eGFR at enrollment (Table 3).

**Table 3:** Estimated relative risk for decline of eGFR ≥ 40% by repeatedly measured albuminuria.

	B	S.E.	EXP (B)	95% CI of Wald		P value
Gender (female)	-0.232	0.772	0.792	-1.746	1.282	0.764
Age (year)	0.011	0.035	1.011	-0.057	0.079	0.749
eGFR (ml/min/1.73 m^2^)	-0.043	0.020	0.976	-0.083	-0.003	0.035
Repeatedly measured albuminuria (per 500mg/day)	0.375	0.107	1.455	0.165	0.585	<0.001

eGFR, estimated glomerular filtration rate; S.E., standard error.

The model using the generalized estimating equations was adjusted for age, sex, and independent factors related to the risk of a decline of eGFR ≥ 40% such as eGFR level during the enrollment period of the trial phase.

### Equality of the repeated measurements of albuminuria to predict the renal outcome

**Fig 2:**
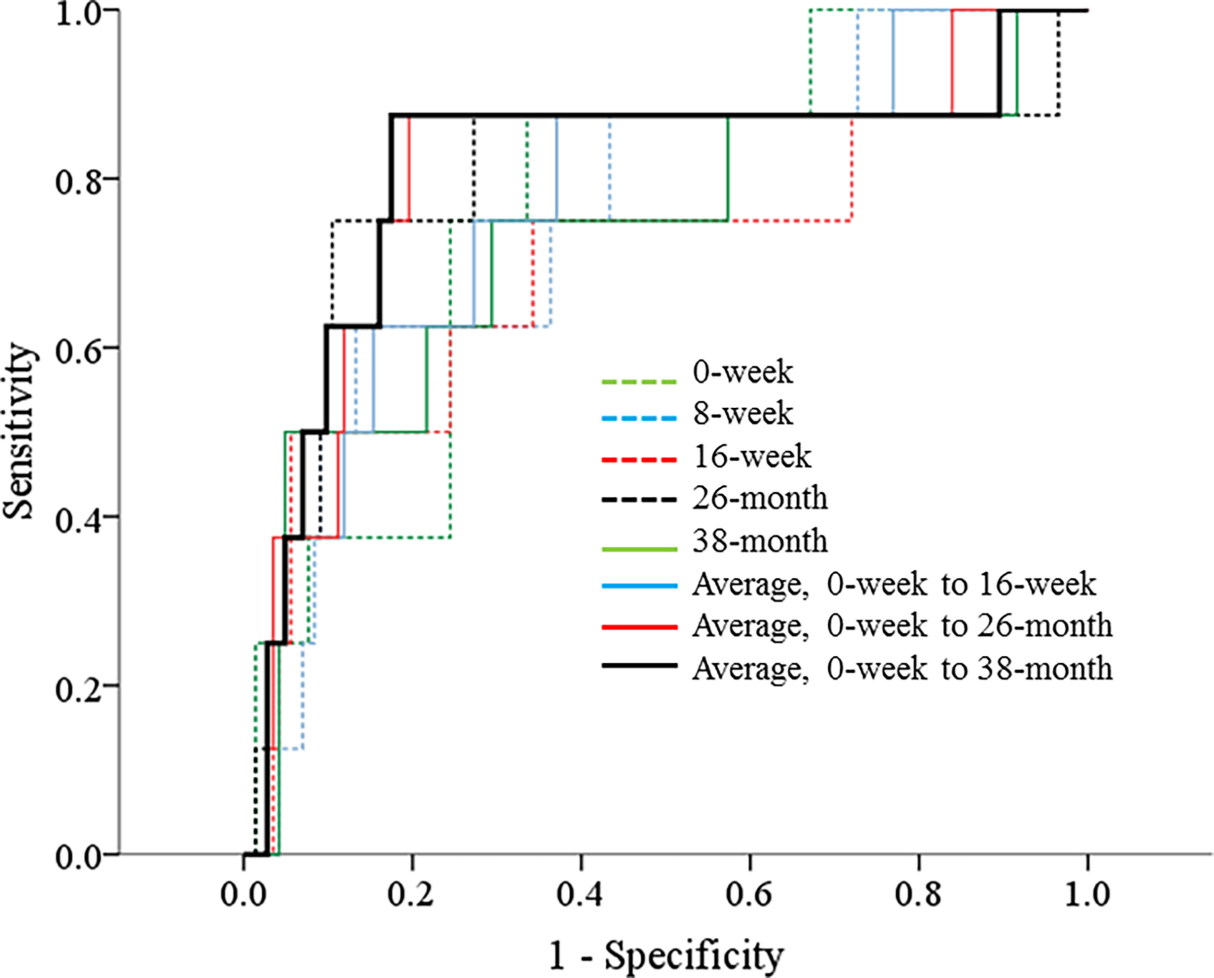
Receiver operating characteristic (ROC) curves with albuminuria at various time points to estimate a decline of estimated glomerular filtration rate ≥ 40%. 0-week, albuminuria measured at the initiation of the trial phase; 8-week, albuminuria measured at 8 weeks after initiation of the trial phase; 16-week, albuminuria measured at 16 weeks after initiation of the trial phase; 26-month, albuminuria measured at the enrollment period of the cohort phase; 38-month, albuminuria measured at the end of the cohort phase; 0-week to 16-week, mean albuminuria measured from the 0-week to the 16-week period of the trial phase; 0-week to 26-month, mean albuminuria measured from the 0-week of the trial phase to the enrollment period of the cohort phase; 0-week to 38-month, mean albuminuria level throughout the entire study period.

To evaluate the ability to predict the renal outcome, we compared the area under the curve (AUC) of ROC among albuminuria values and mean albuminuria values (Table 4 and Fig 2). The AUC was the highest in the ROC curve using the mean albuminuria value during the entire study period to estimate the renal outcome (Table 4). However, the AUC by the mean albuminuria level during the entire study period was not significantly different from the AUC values of the other albuminuria measurements using a test of equality of ROC areas (Table 4). With a cut-off value of 969 mg/day albuminuria during the enrollment period that was measured before an ARB treatment, a decline of eGFR ≥ 40% was predicted with a sensitivity of 88.9% and specificity 65.8% (S3 Table). With a cut-off value of 897 mg/day for mean albuminuria at 38.1 ± 4.1 months after an ARB treatment, a decline of eGFR ≥ 40% was predicted with a sensitivity of 88.9% and specificity of 81.3%.

**Table 4:** AUC of parameters of albuminuria measured to predict a decline of eGFR ≥ 40%.

	AUC	95% CI of AUC		P value of AUC	P value*
Albuminuria (mg/day)					
0-week	0.769	0.622	0.916	0.011	0.392
8-week	0.757	0.593	0.921	0.015	0.311
16-week	0.701	0.480	0.922	0.056	0.148
26-month	0.797	0.581	1.000	0.005	0.703
38-month	0.727	0.517	0.938	0.031	0.203
Averaged albuminuria (mg/day)					
0-week to 16-week	0.776	0.608	0.945	0.009	0.374
0-week to 26-month	0.809	0.631	0.988	0.003	0.893
0-week to 38-month	0.812	0.616	1.000	0.003	-

AUC, area under the curve; eGFR, estimated glomerular filtration rate; CI, confidence interval.

*P value estimates probability of difference of AUC with the measurements of albuminuria at various time points to estimate a decline of eGFR ≥ 40% during the entire study period compared to that of the mean albuminuria throughout the entire study period.

0-week, albuminuria measured at the initiation of the trial phase; 8-week, albuminuria measured at 8 weeks after initiation of the trial phase; 16-week, albuminuria measured at 16 weeks after initiation of the trial phase; 26-month, albuminuria measured at the enrollment period of the cohort phase; 38-month, albuminuria measured at the end of the cohort phase; 0-week to 16-week, mean albuminuria measured from the 0-week to the 16-week period of the trial phase; 0-week to 26-month, mean albuminuria measured from the 0-week of the trial phase to the enrollment period of the cohort phase; 0-week to 38-month, mean albuminuria level throughout the entire study period.

## Discussion

We analyzed the ability of repeatedly measured albuminuria to predict a renal outcome in non-diabetic hypertensive CKD patients. All measurements of albuminuria at different time periods and mean albuminuria values at different time periods were independent risk factors for a worse renal outcome and equally effective at predicting the outcome, although the estimation tended to be more effective using the mean albuminuria level at the 38-month follow-up time point.

The US Food and Drug Administration currently accepts changes in eGFR as a surrogate endpoint for the development of kidney failure in clinical trials of kidney disease progression [19]. A scientific workshop sponsored by the National Kidney Foundation and the US Food and Drug Administration recommended that an eGFR decline ≥ 40% is more broadly accepted than a 30% decline across a wider range of baseline GFR values and patterns of treatment effects on GFR [19]. Therefore, we chose the criterion of renal outcome as the recommendation. We also analyzed the data using criteria of renal outcomes such as a 30% decline in eGFR and decline in a slope of eGFR ≥ 4 mL/min/1.73 m^2^/year and found the results were as similar to those in this manuscript (data not shown).

In diabetic CKD, basal albuminuria is the strongest predictor of well-known baseline risk factors for a renal outcome and change in proteinuria during the first 6 months and residual albuminuria at 6 months after RASi treatment is also associated with a renal outcome even with adjustment for basal proteinuria [2, 8]. Studies including IgA nephropathy and similar findings were reported [1, 10]; additionally, time-averaged proteinuria is the most important prognostic indicator in favor of basal proteinuria, residual proteinuria, and changes in proteinuria during follow-up [1, 10]. Among CKD patients, baseline proteinuria is also a well-known risk factor for renal outcome [3, 4, 20, 21]. A Chinese cohort study showed residual proteinuria at 6 month after RASi treatment and time-averaged proteinuria > 1 g/day during the entire follow-up period remained the independent predictors for renal endpoints after adjustment for basal proteinuria [3]. Residual proteinuria at 3 months after RASi treatment showed higher sensitivity and specificity to predict a renal outcome than did changes in proteinuria [4]. However, all studies mentioned were not able to remove the interaction between baseline proteinuria and residual proteinuria, changes in proteinuria, time-averaged proteinuria, or response to RASi treatment according to basal proteinuria level because all proteinuria measurements were included in the regression model, which assumes linearity, independency, homoscedasticity, and normality of residuals between dependent and independent variables. However, repeatedly measured parameters after treatment are not independent from each other and the general regression model cannot remove interactions between variables. Therefore, we used GEE, which is suitable to analyze the data with repeatedly measured variables with dependency.

We evaluated the ability of albuminuria values to estimate a renal outcome and showed the time-averaged albuminuria at 38 months was better able to predict a renal outcome, although the difference was not statistically significant. From IgA nephropathy data, performance of the regression model with time-averaged proteinuria to estimate the deterioration in eGFR slope was rapidly improved during 2–3 years after RASi treatment and then plateaued over the remaining time frames, and the author suggested that 24- and 36-month observations for deteriorating renal function would be suitable because the outcome was predicted 50% sooner at a loss of only 25% accuracy compared with the longer observation period [10]. Therefore, the 38-month observation period of this study would be suitable for estimating the renal outcome. In this model, the ROC area of proteinuria to estimate a renal outcome was better than that of the Ramipril Efficacy In Nephropathy (REIN) study [4]. The AUC values of albuminuria measurements here were 0.701–0.812, while those of proteinuria measurements in the REIN study were 0.63 and 0.58 [4]. This difference might result from the different outcome criteria and differences in power to predict renal outcomes between proteinuria and albuminuria. We showed that the cut-off point of albuminuria to predict a decline of eGFR ≥ 40% was 900 mg/day of time-averaged albuminuria at 38 months with 88.9% sensitivity and 81.3% specificity. However, additional studies are needed to confirm the power of predictability of these criteria in CKD patients.

This study has several limitations worth noting. First, we did not include all participants in the trial phase; nevertheless, the inclusion rate in this cohort study was similar to that of other cohort studies such as the observational TOHP study. Second, the participants enrolled in this study were more compliant with ARB treatment and more frequently allocated to the conventional education group in the trial phase, which might be a confounding factor affecting the study’s final results. Third, there was a confounding effect of amount of salt intake derived from the 24-hour urine sodium measurement modulated by diet education to evaluate the effect of albuminuria measured at 8 and 16 weeks on a decline of eGFR at 38 months. As we showed in our earlier report [15], intensive low-salt diet education affects the decline in albuminuria and salt intake. Actually, the effect of low-salt diet education or salt intake amount on the renal outcome overcame that of albuminuria at 8 and 16 weeks on logistic regression. However, the difference in changes of albuminuria and salt intake according to the low-salt diet education vanished during the cohort and entire study period and did not influence the relationship between albuminuria and renal outcome in other time points. Finally, we could not exclude errors in measuring albuminuria and sodium through 24-hour urine collection, although we tried to remove it by using the standardized urine creatinine equation.

In conclusion, the ability of albuminuria to predict renal events did not differ at any time point in CKD patients. The time-averaged albuminuria cut-off of 900 mg/day during the 3-year follow-up period showed high sensitivity and specificity for predicting a worse renal outcome in CKD patients.

## Supporting information

S1 Table Characteristics according to participation during the enrollment period of the trial phase. (DOCX)

S2 Table Difference of albuminuria during study period. (DOCX)

S3 Table Cut-off value of albuminuria to predict the decline of eGFR ≥ 40% during 38.1 ± 4.1 months by ROC curve. (DOCX)

S1 File Individual patient information. (XLSX)
