# Supplementary material for: Albuminuria during treatment with angiotensin type II receptor blocker is a predictor for GFR decline among non-diabetic hypertensive CKD patients
Source: PLoS One. 2018 Aug 27;13(8):e0202676. doi: 10.1371/journal.pone.0202676 (PMC6110474; doi:10.1371/journal.pone.0202676)
Supplement: S1 Table — (DOCX) [file pone.0202676.s001.docx]

**S1 Table. Characteristics according to participation during the enrollment period of the trial phase**

|  | Non-participants in this analysis (n=70) | Participants in this analysis (n=165) | p-value |
| --- | --- | --- | --- |
| Demographics |  |  |  |
| Age (years) | 50.0 ± 13.8 | 50.0 ± 12.9 | 0.994 |
| Gender (Male) | 38 (54.3 %) | 79 (47.9 %) | 0.369* |
| Weight (kg) | 68.3 ± 15.5 | 67.9 ± 12.4 | 0.832 |
| Height (cm) | 164 ± 9 | 163 ± 9 | 0.764 |
| Systolic blood pressure (mmHg) | 131 ± 12 | 131 ± 12 | 0.384 |
| Diastolic blood pressure (mmHg) | 79 ± 9 | 79 ± 9 | 0.924 |
| Life style |  |  |  |
| Smoker never/ex/current | 46/13/11 | 120/29/16 | 0.383* |
| Drinker never/ex/current | 32/10/28 | 89/11/65 | 0.146* |
| Blood measurement |  |  |  |
| WBC (/mm^3^) | 6238 ± 1493 | 6475 ± 1844 | 0.342 |
| hemoglobin (g/dL) | 14.2 ± 1.8 | 13.8 ± 1.7 | 0.075 |
| Hematocrit (%) | 41.7 ± 4.7 | 40.5 ± 4.5 | 0.072 |
| Platelet (/mm^3^) | 125 ± 64 | 241 ± 58 | 0.456 |
| Creatinine (mg/dL) | 1.16 ± 0.43 | 1.14 ± 0.40 | 0.699 |
| eGFR (ml/min/1.73 m^2^) | 66.5 ± 22.8 | 67.1 ± 24.7 | 0.859 |
| Urine measurement |  |  |  |
| e24-h urine creatinine (mg/day) | 1312 ± 365 | 1299 ± 298 | 0.789 |
| e24-h urine Na (mEq/day) | 170 ± 78 | 170 ± 73 | 0.395 |
| e24-h urine albumin (mg/day) | 960 ± 1073 | 1061 ± 1094 | 0.516 |
| Creatinine clearance (ml/min) | 81.2 ± 35.6 | 80.0 ± 32.1 | 0.812 |
| eProtein intake (g/kg/day) | 0.87 ± 0.52 | 0.94 ± 0.46 | 0.283 |
| Comorbidities |  |  |  |
| Dyslipidemia | 40 (59.7 %) | 92 (58.2 %) | 0.837* |
| Coronary artery disease | 1 (1.4 %) | 1 (0.6 %) | 0.510† |
| Stroke | 2 (2.9 %) | 4 (2.4 %) | 1.000† |
| Medications before the trial phase |  |  |  |
| Angiotensin converting enzyme blocker | 8 (11.4 %) | 9 (5.5 %) | 0.106* |
| Angiotensin II receptor blocker | 50 (71.4 %) | 129 (78.2 %) | 0.266* |
| beta-Blocker | 8 (11.4 %) | 39 (23.6 %) | 0.032* |
| Calcium channel blocker | 36 (51.4 %) | 90 (54.5 %) | 0.661* |
| Diuretics | 9 (12.9 %) | 19 (11.5 %) | 0.772* |
| Other hypertension medications | 2 (2.9 %) | 15 (9.1 %) | 0.105† |
| anti-lipid medications | 32 (45.7 %) | 87 (52.7 %) | 0.325* |
| anti-platelet medications | 26 (37.1 %) | 79 (47.9 %) | 0.130* |
| Steroids | 1 (1.4 %) | 6 (3.6 %) | 0.667† |
| Immunosuppressive agents | 2 (2.9 %) | 2 (1.2 %) | 0.585† |
| Nonsteroidal anti-inflammatory drugs | 3 (4.3 %) | 5 (3.0 %) | 0.698† |
| Compliance to ARB medication (%, mean ± SD) | 93.1 ± 8.7 | 95.5 ± 7.6 | 0.033 |
| Allocation to intensive education group | 46 (65.7 %) | 74 (44.8 %) | 0.003* |

WBC: white blood cell, eGFR: estimated glomerular filtration rate calculated by MDRD equation using IDMS-traceable serum creatinine, e24-h urine Na: Estimated 24-hour urine sodium excretion by adjustment of measured 24-hour urine sodium based on the estimated daily creatinine amount by Tanaka’s equation. e24-h urine albumin: Estimated 24-hour urine albumin excretion by adjustment of measured 24-hour urine albumin based on the estimated daily creatinine amount by Tanaka’s equation. eProtein intake: Estimated daily protein intake calculated from adjusted 24-hour urine urea nitrogen based on the estimated daily creatinine amount by Tanaka’s equation, ARB: Antiotensin II receptor blocker, SD: standard deviation. Medications: used before enrollment, Compliance to ARB medication: calculated at 16-week trial period.

Parametric variables were expressed as mean ± standard deviation. Comparisons were made using Pearson’s chi-square test or Fisher’s exact test for categorical variables and the independent Student’s t test for continuous variables.

* Pearson’s chi-square test, † Fisher’s exact test
