# Supplementary material for: Albuminuria during treatment with angiotensin type II receptor blocker is a predictor for GFR decline among non-diabetic hypertensive CKD patients
Source: PLoS One. 2018 Aug 27;13(8):e0202676. doi: 10.1371/journal.pone.0202676 (PMC6110474; doi:10.1371/journal.pone.0202676)
Supplement: S2 Table — (DOCX) [file pone.0202676.s002.docx]

**S2 Table. Difference of albuminuria during study period**

|  | 0-week | 8-week | 16-week | 26-month | 38-month |
| --- | --- | --- | --- | --- | --- |
| Number | 165 | 162 | 164 | 162 | 157 |
| Mean (mg/day) | 1061.06 | 480.67 | 593.93 | 621.56 | 643.18 |
| Standard deviation | 1094.99 | 702.26 | 836.78 | 908.65 | 930.47 |
| Minimum (mg/day) | 25.06 | 11.73 | 14.61 | 8.97 | 8.45 |
| Maximum (mg/day) | 5499.77 | 6484.39 | 5568.29 | 7686.66 | 5567.08 |
| Range (mg/day) | 5474.71 | 6472.66 | 5553.67 | 7677.69 | 5558.63 |

0-week: albuminuria measured at the initiation of trial-phase, 8-week: albuminuria measured at 8 weeks after initiation of trial-phase, 16-week: albuminuria measured at 16 weeks after initiation of trial-phase, 26-month: albuminuria measured at the enrollment period of cohort-phase, 38-month: albuminuria measured at the end of cohort-phase,
