# Supplementary material for: Albuminuria during treatment with angiotensin type II receptor blocker is a predictor for GFR decline among non-diabetic hypertensive CKD patients
Source: PLoS One. 2018 Aug 27;13(8):e0202676. doi: 10.1371/journal.pone.0202676 (PMC6110474; doi:10.1371/journal.pone.0202676)
Supplement: S3 Table — (DOCX) [file pone.0202676.s003.docx]

**S3 Table. Cut-off value of albuminuria to predict the decline of eGFR ≥ 40% during 38.1 ± 4.1 months by ROC curve**

|  | Cut-off value | Sensitivity | Specificity | PPV | NPV |
| --- | --- | --- | --- | --- | --- |
| Albuminuria (mg/day) |  |  |  |  |  |
| 0-week | 969 | 88.9 | 65.8 | 13.1 | 99.0 |
| 8-week | 992 | 66.7 | 87.7 | 24.0 | 97.8 |
| 16-week | 632 | 66.7 | 75.7 | 14.0 | 97.5 |
| 26-month | 1165 | 77.8 | 89.5 | 30.4 | 98.6 |
| 38-month | 612 | 75.0 | 70.5 | 12.0 | 98.1 |
| Averaged albuminuria (mg/day) | |  |  |  |  |
| 0-week to 16-week | 756 | 77.8 | 72.2 | 14.3 | 98.2 |
| 0-week to 26-month | 897 | 88.9 | 79.2 | 20.5 | 99.2 |
| 0-week to 38-month | 897 | 88.9 | 81.3 | 21.6 | 99.2 |

PPV: positive predictive value, NPV: negative predictive value, 0-week: albuminuria measured at the initiation of trial-phase, 8-week: albuminuria measured at 8 weeks after initiation of trial-phase, 16-week: albuminuria measured at 16 weeks after initiation of trial-phase, 26-month: albuminuria measured at the enrollment period of cohort-phase, 38-month: albuminuria measured at the end of cohort-phase, 0-week to 16-week: average of albuminuria measured from 0-week period to 16-week period of trial-phase, 0-week to 26-month: average of albuminuria measured from 0-week period of trial phase to enrollment period of cohort-phase, 0-week to 38-month: average of albuminuria throughout whole study period
